# Thrombin generation abnormalities in commonly encountered platelet function disorders

**DOI:** 10.1111/ijlh.13638

**Published:** 2021-06-29

**Authors:** Tanmya Sharma, Justin G. Brunet, Subia Tasneem, Stephanie A. Smith, James H. Morrissey, Catherine P.M. Hayward

**Affiliations:** ^1^ Department of Pathology and Molecular Medicine McMaster University Hamilton ON Canada; ^2^ University of Michigan Medical School Ann Arbor MI USA; ^3^ Department of Medicine McMaster University Hamilton ON Canada; ^4^ Hamilton Regional Laboratory Medicine Program Hamilton ON Canada

**Keywords:** bleeding, blood platelet disorders, coagulation, platelet storage pool deficiency, thrombin generation

## Abstract

**Introduction:**

Studies of thrombin generation (TG) with platelet‐rich plasma (PRP) and platelet‐poor plasma (PPP) have provided insights on bleeding disorders. We studied TG for a cohort with commonly encountered platelet function disorders (PFD).

**Methods:**

Participants included 40 controls and 31 with PFD due to: nonsyndromic dense granule (DG) deficiency (PFD‐DGD, n = 9), *RUNX1* haploinsufficiency (n = 6) and aggregation defects from other, uncharacterized causes (n = 16). TG was tested with PRP and PPP samples. As DG store ADP and polyphosphate that enhance platelet‐dependent TG, PFD‐DGD PRP TG was tested for correction with ADP, polyphosphate and combined additives. Tissue factor pathway inhibitor (TFPI), platelet factor V (FV), and platelet *TFPI* and *ANO6* transcript levels were also evaluated. Findings were tested for associations with TG endpoints and bleeding.

**Results:**

PFD samples had impaired PRP TG, but also impaired PPP TG, with strong associations between their PRP and PPP TG endpoints (*P* ≤ .005). PFD‐DGD PRP TG endpoints showed associations to PPP TG endpoints but not to DG counts, and were improved, but not fully corrected, by adding polyphosphate and agonists. PFD participants had increased plasma TFPI and reduced platelet TFPI (*P* ≤ .02) but normal levels of platelet FV, and platelet *TFPI* and *ANO6* transcripts levels. PFD plasma TFPI levels showed significant association to several PPP TG endpoints (*P* ≤ .04). Several PFD PRP TG endpoints showed significant associations to bleeding symptoms, including wound healing problems and prolonged bleeding from minor cuts (*P* ≤ .04).

**Conclusion:**

TG is impaired in commonly encountered PFD, with their PRP TG findings showing interesting associations to symptoms.

## 1 INTRODUCTION

Platelets have an important role in hemostasis and when activated, promote thrombin generation (TG).^1^, ^2^ Many of the commonly encountered platelet function disorders (PFD) are nonsyndromic disorders that present with symptoms of mucocutaneous and challenge‐related bleeding, and laboratory findings of impaired aggregation responses to multiple agonists and/or platelet dense granule deficiency (DGD).^3^ While some of these PFD are caused by pathogenic *RUNX1* mutations (PFD‐RUNX1), others are caused by uncharacterized molecular defects that manifest with nonsyndromic DGD (PFD‐DGD) or impaired aggregation responses to multiple agonists (PFD‐OTH).^3^ At present, there are uncertainties about how these PFD affect TG.

We postulated that TG could be abnormal in commonly encountered PFD, based on several lines of evidence. First, platelet aggregation influences TG^4^ and in some PFD (including PFD‐RUNX1), platelet activation and aggregation are impaired.^3^, ^5^ Second, TG by normal platelets is increased by platelet‐activating agonists.^6^ Third, platelet DG store and release constituents that affect TG, including ADP that enhances TG by promoting platelet activation and polyphosphate that directly accelerates TG.^1^, ^7^, ^8^ Finally, PFD samples drawn after desmopressin therapy show improved platelet‐dependent TG,^9^ although the study that reported this interesting finding did not report if basal TG was impaired in PFD.

Calibrated automated thrombograms (CAT) have proved useful to assess TG by both platelet‐poor plasma (PPP) and platelet‐rich plasma (PRP) samples from patients with a variety of bleeding disorders including several inherited and acquired PFD, hemophilia and other factor deficiencies.^10^, ^11^, ^12^, ^13^, ^14^, ^15^ Accordingly, we used CAT with PRP and PPP to conduct a pilot study of a well‐characterized, PFD cohort, with increased risks for mucocutaneous and challenge‐related bleeding.^3^ This cohort included persons with PFD‐DGD, PFD‐RUNX1, and PFD‐OTH.^3^, ^16^ We also assessed other parameters that are known to influence TG, including (a) tissue factor pathway inhibitor (TFPI)^17^, ^18^ which influences TG in Quebec platelet disorder (QPD),^14^ coagulation factor deficiencies,^17^, ^19^ and bleeding of unknown cause (BUC)^20^; (b) platelet factor V (FV), which is stored in α‐granules and is reduced in some PFD^14^, ^21^; and (c) platelet *ANO6/PPIA* transcript ratios as *ANO6* mutations cause a severe platelet procoagulant defect known as Scott's syndrome.^22^, ^23^ We postulated that characterizing *ANO6* expression in our cohort might provide new insights as PFD‐RUNX1 are known to alter the expression of many genes.^3^, ^24^ Finally, we looked for potential relationships between findings and bleeding, using data on symptoms and bleeding scores for our PFD cohort.^3^

## 2 METHODS

The study was carried out in accordance with the revised Helsinki protocol, with approval by the Hamilton Integrated Research Ethics Board (HiREB project 12‐149) and written informed consent of all participants.

### 2.1 Participants

PFD participants were part of a consecutive case cohort, recruited to the study at Hamilton Health Sciences, Ontario, Canada during the period between September 2012 and March 2018.^3^ Samples for TG studies were collected between January 2018 and April 2021. Participant identities were anonymized at sample collection.

The cohort included: index cases and additional, affected family member participants (“non‐index cases”) to limit bias; each was confirmed to have DGD and/or aggregation defects.^3^ Six persons in the initial PFD cohort were not evaluated due to: very young age/size (n = 2); anticoagulant therapy (n = 1); lost to follow up (n = 2); or moved far away (n = 1). Two participants that provided TG samples declined further donations during the COVID‐19 pandemic. Table S1 lists the PFD cohort participants, and the tests performed on their samples.^3^ Reference^3^ contains details of: the initial inclusion and exclusion criteria; diagnostic test findings (such as platelet counts, maximal aggregation responses, DG numbers); bleeding symptoms and International Society on Thrombosis and Haemostasis‐Bleeding Assessment Tool [ISTH‐BAT] scores; exome sequence findings; and transcript levels for RUNX1‐regulated genes for PFD‐RUNX1 participants. The 31 PFD participants evaluated in the present study (ages as median [range], % female, number of families [range of affected individuals studied/family]) included n = 9 with PFD‐DGD (63 [31‐81] years; 89% females; 5 families [1‐2/family]; average DG count/platelet: 1.7 [0.9‐2.3], lower limit of reference interval: 4.9 DG/platelet^25^); n = 6 with PFD‐RUNX1 (48 [22‐73] years; 50% females; 5/6 from one family); and n = 16 with PFD‐OTH (51 [20‐80] years; 69% females; 5 families [1‐4/family]).^3^, ^16^ All had normal coagulation screening tests (INR/PT, APTT, fibrinogen, thrombin time) and none had von Willebrand disease or FVIII deficiency.^3^

PFD and control samples were tested in parallel. Controls included 20 previously reported general population controls (age and sex‐matched to PFD participants) whose TG findings were included in our study of TG in QPD,^14^ and 20 additional general population controls.

### 2.2 Sample preparation

Blood (20‐65 mL) was collected by venipuncture and anticoagulated with 0.109 mol L^−1^ buffered sodium citrate (1:9 volume/volume) for preparation of PPP and PRP for TG, and for assays of plasma TFPI, as described^14^; and with acid citrate dextrose (ACD, 1:6 volume/volume with [final concentrations, in blood] 1 mmol L^−1^ theophylline and 3 µmol L^−1^ PGE_1_ [Sigma Aldrich Canada]) for preparation of platelet lysates (for TFPI and FV determinations) and leukodepleted platelet pellets for RNA isolation, as described.^14^, ^26^, ^27^

### 2.3 Thrombin generation

Samples from general population controls (some donated multiple times)^14^ were included on each run. TG was evaluated by CAT,^14^ as recommended,^11^ with PRP adjustment to 150 × 10^9^ platelets/L with autologous plasma. Testing was completed within 3‐4 hours of blood collection.

TG was quantified using a Fluoroskan plate reader (Thermo Fisher Scientific) and Thrombinoscope reagents and software (Stago Canada Ltd.).^14^ PPP test wells contained: 80 μL PPP and 20 μL PPP reagent (containing 4 μmol L^−1^ phospholipids, 5 pM TF).^14^ PRP test wells contained: 80 μL PRP and 20 μL PRP reagent (containing 0.5 pM TF).^14^ TG was started by dispensing 20 μL of FluCa reagent into each well.^14^ TG measurements (mean of triplicate determinations, taken at 20‐second intervals for 60 minutes at 37°C) were used to estimate endogenous thrombin potential (ETP, nmol L^−1^*min), peak thrombin concentration (nmol L^−1^), time to peak TG (min), and lag time (min).^11^, ^14^

Because prior studies on TG in PFD‐DGD tested the correcting effects of ADP^8^ or polyphosphate,^1^ but not their combined effects, PRP from sixteen representative controls, and from seven representative PGD‐DGD participants, was used to test TG with and without additives. Briefly, samples were tested after a 10 minute PRP preincubation with or without (described with final concentrations): 10 µmol L^−1^ ADP (Sigma‐Aldrich Canada; tested with 12/16 controls); stronger, combined agonist stimulation with 10 µmol L^−1^ ADP, 50 µmol L^−1^ of the human PAR1 receptor activating peptide SFLLRN (Bachem Bioscience Inc, Philadelphia, PA, USA) and 10 µg/mL Horm collagen (Helena Laboratories, Beaumont, TX, USA); or 10 µg/mL polyphosphate (monomer concentration of natriumpolyphosphate P70, provided to the Morrissey laboratory by Dr Thomas Staffel, BK Giulini), tested with or without combined agonist stimulation.

### 2.4 Assessments of transcript levels in platelets

RNA was isolated from leukodepleted platelets and reverse transcribed using a high‐capacity RNA‐to‐cDNA kit (Applied Biosystems, Thermo Fisher Scientific), as described,^26^ followed by digital droplet polymerase chain reaction (ddPCR) analyses of transcripts levels for *ANO6* (probe Hs03805835_m1, FAM label; Life Technologies, Thermo Fisher Scientific), and *TFPI* (probe Hs00409207_m1 FAM label; Life Technologies, Thermo Fisher Scientific), relative to copies of *PPIA* (probe Hs99999904_m1, VIC label; Life Technologies, Thermo Fisher Scientific), similar to methods described.^26^

### 2.5 Enzyme‐linked immunoassays

Platelet and plasma TFPI (monoclonal antibody capture, polyclonal antibody detection; validated by the supplier to detect both natural and recombinant TFPI), and platelet FV, were quantified by enzyme‐linked immunoassay (ELISA) using methods previously described, with comparison to previously reported^14^ and additional controls.

### 2.6 Statistical analyses

Paired PRP and PPP, and/or first sample data for those with multiple determinants, were analyzed. Based on the final number of subjects evaluated, and pooled standard deviations for TG endpoints, power calculations estimated that the study had an ≥80% chance of detecting significant differences in TG between PFD and control subjects, recognizing that findings might be heterogenous.

Two‐tailed Mann‐Whitney tests, with Bonferroni correction for multiple comparisons, were used to assess differences in TG endpoints, protein, and transcript levels. Wilcoxon signed‐rank test was used to assess the effects of additives on TG endpoints. Linear regression was used to assess relationships between (a) PPP and PRP TG endpoints; (b) protein levels and TG endpoints; (c) transcript levels and PRP TG endpoints; and (d) ISTH‐BAT scores and findings for TG and other laboratory assays. Relationships between laboratory findings and bleeding symptoms were further explored using Mann‐Whitney tests to compare the laboratory findings for PFD participants that did or did not report: bruising without trauma, bruising disproportionate to trauma, prolonged wound bleeding, wound healing problems, prolonged menses, or challenge‐related bleeding (with surgery, dental procedures, trauma and/or childbirth).

## 3 RESULTS

### 3.1 Thrombin generation findings and their association to bleeding

**FIGURE 1:**
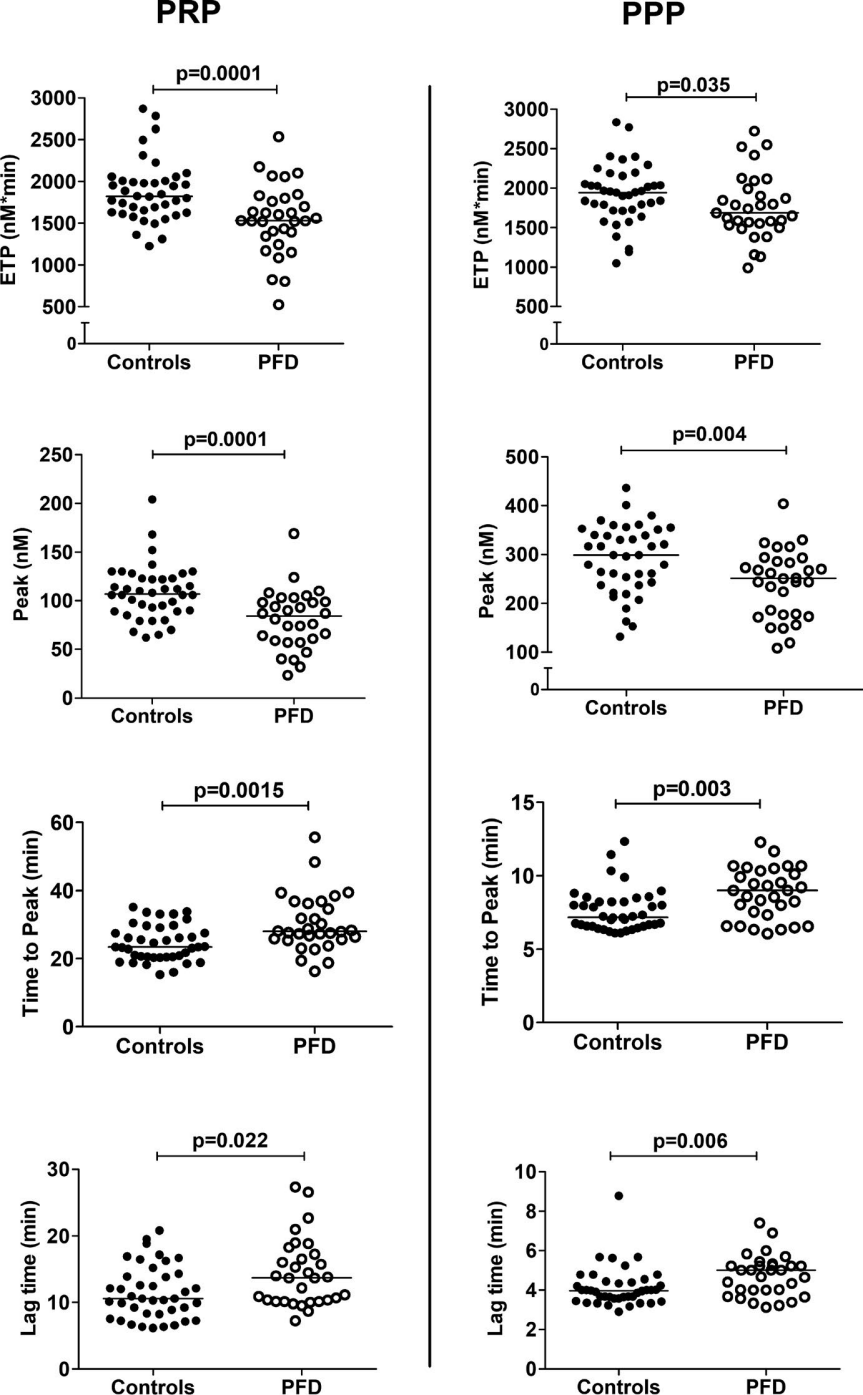
Thrombin generation findings for platelet function disorder and control samples, evaluated using platelet‐rich and platelet‐poor plasma. Panels compare platelet‐rich plasma (PRP) and platelet‐poor plasma (PPP) data for general population controls (n = 40) and platelet function disorder (PFD) participants (n = 31) for the endpoints of: endogenous thrombin potential (ETP, nmol L^−1^*min), peak thrombin concentration (Peak, nmol L^−1^), time to peak thrombin generation (Time to Peak, min), and lag time (min) for samples. Horizontal lines indicate medians. Significant *P* values are indicated

TG endpoints were similar for index and nonindex PFD cases (*P* ≥ .19), and for males and females (*P* ≥ .22), and showed no association to age (*R* ^2^ ≤ .01; *P* ≥ .39). The median values for all PRP and PPP TG endpoints were significantly different for PFD compared to control samples (*P* ≤ .03) (Figure 1). Both PFD PRP and PPP had a significantly reduced median ETP and peak thrombin concentration, and prolonged median lag time and time to peak TG, relative to control samples, with some overlap in PFD and control findings (Figure 1). Both PFD and control samples showed strong associations between their PRP and PPP TG endpoints (R^2^ 0.24‐0.67; *P* ≤ .005; Figure. S1).

**FIGURE 2:**
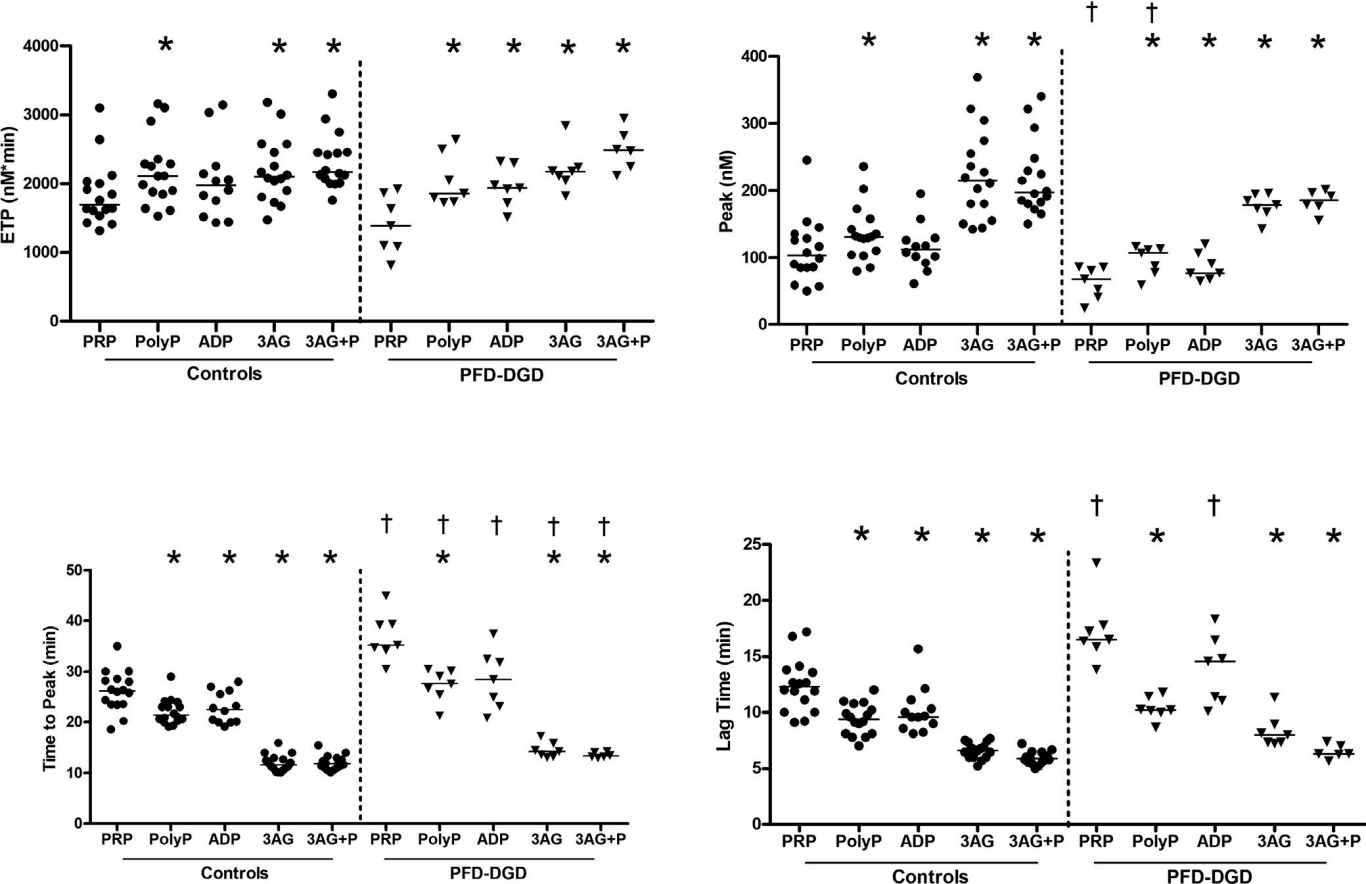
Thrombin generation endpoints of platelet‐rich plasma from nonsyndromic dense granule deficient and control participants, tested with and without polyphosphate and other additives. The thrombin generation endpoints measured included endogenous thrombin potential (ETP, nmol L^−1^*min), peak thrombin concentration (Peak, nmol L^−1^), time to peak thrombin generation (Time to Peak, min), and lag time (min) for: n = 16 healthy general population controls (12/16 tested with ADP) and n = 7 individuals with nonsyndromic dense granule deficiency (PFD‐DGD). Samples were supplemented with either polyphosphate (“PolyP”), ADP, multiple agonists (3AG), or multiple agonists and PolyP (3AG+P). One PFD‐DGD sample was not tested with 3AG+P. Medians are indicated by horizontal lines. *indicate significant differences in thrombin generation endpoints (*P* < .05) for samples tested with versus without additives. † indicate significant differences in thrombin generation endpoints (*P* < .05) for PFD‐DGD compared to control samples (*P* < .05)

Among PFD‐DGD participants, DG counts did not show significant associations to PRP TG endpoints (*R* ^2^.001‐.20, *P* ≥ .23), however, many had fairly similar reductions in DG counts (Table S1). Without additives, PFD‐DGD PRP showed significantly prolonged time to peak TG (*P* = .0005), prolonged lag time (*P* = .002), and reduced peak thrombin concentration (*P* = .01), but normal ETP, relative to control samples (Figure 2). Polyphosphate, and all additives except ADP, significantly increased the ETP and peak thrombin concentration and significantly shortened time to peak TG and lag time of control PRP (*P* ≤ .03; Figure 2). All additives significantly increased the ETP and peak thrombin concentration, and significantly shortened time to peak TG and lag time of PFD‐DGD PRP, with the exception that ADP did not significantly shorten the time to peak TG or lag time (*P* ≤ .03; Figure 2). Most control and PFD‐DGD PRP TG endpoints were not significantly different from each other in tests with additives, however, the time to peak TG for PFD‐DGD remained prolonged with all additives, and the lag time of PFD‐DGD PRP remained prolonged with added ADP (Figure 2). Significant associations between PFD‐DGD PRP and PPP peak thrombin concentrations (*R* ^2^ .69, *P* = .006; Figure. S2), suggested that their plasma influenced their PRP TG endpoints.

### 3.2 Protein and transcript levels and their association to bleeding

**FIGURE 3:**
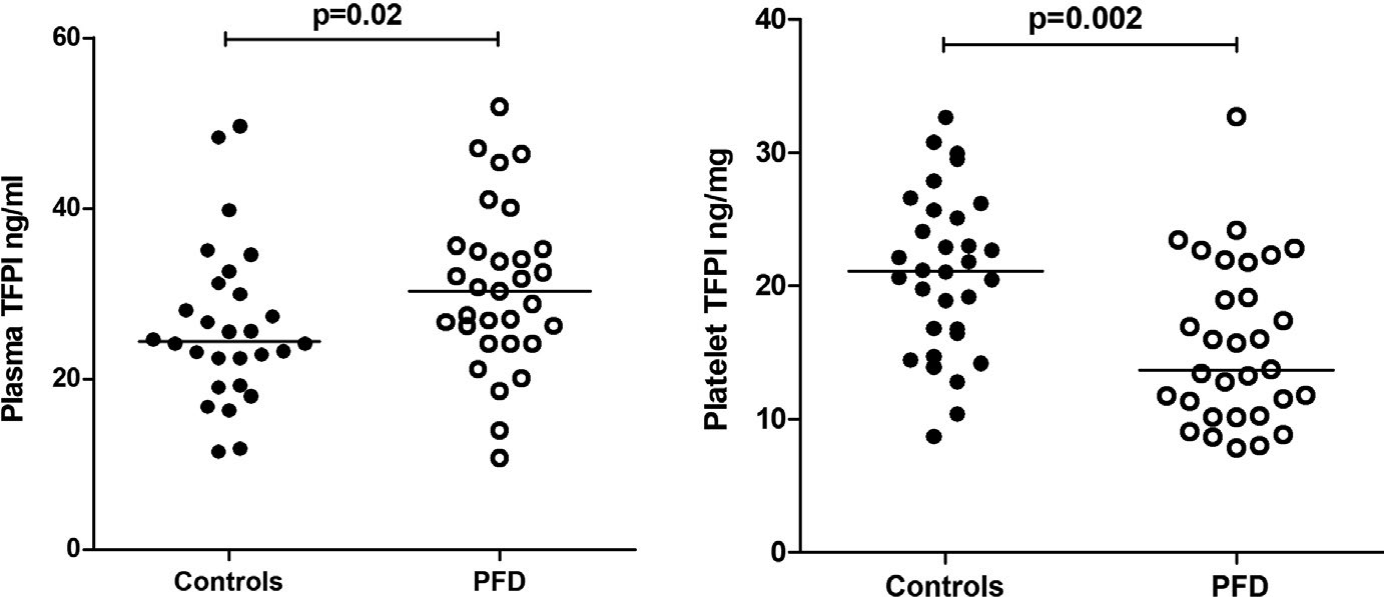
Platelet and plasma levels of tissue factor pathway inhibitor for platelet function disorder and control participants. *P* values indicate significant differences in the levels of plasma and platelet tissue factor pathway inhibitor (TFPI) for platelet function disorder (PFD) compared to control participants

Relative to controls, PFD participants had significantly higher median plasma TFPI levels and significantly lower platelet TFPI levels (*P* ≤ .02; Figure 3). Plasma TFPI levels of PFD participants showed weak but significant positive associations to the PPP TG endpoints time to peak (R^2^ 0.13; *P* = .04) and lag time (*R* ^2^ .21; *P* = .009), but not to PRP TG endpoints (*R* ^2^ ≤ .05; *P* ≥ .21) (Table S2). Platelet TFPI levels of PFD participants did not show significant association to PRP TG endpoints (*R* ^2^ ≤ .10; *P* ≥ .08) (Table S2). Platelet *TFPI/PPIA* transcript ratios for PFD participants were not significantly different from controls (Table 1) and did not show significant association to PFD PRP TG endpoints (*R* ^2^ ≤ .13; *P* ≥ .05)(Table S2). Platelet *ANO6/PPIA* transcript ratios and platelet factor V levels were normal in PFD participants (Table 1), and did not show significant associations to PRP TG endpoints (*R* ^2^ ≤ 0.13; *P* ≥ .06) (Table S2).

**TABLE 1:** Comparisons of the platelet levels of *TFPI* and *ANO6* transcripts and factor V antigen for controls and platelet function disorder participants

Parameter	PFD participants	Controls	*P* value
*TFPI/PPIA* transcript ratios	0.4 [0.2‐0.6], n = 31	0.4 [0.3‐1.0], n = 23	.54
*ANO6/PPIA* transcript ratios (X1000)	1.6 [0.8‐2.5], n = 31	1.6 [0.8‐3.4], n = 23	.11
Platelet factor V (µg/mg total protein)	0.8 [0.2‐2.6], n = 31	0.9 [0.2‐1.7], n = 29	.59

Note

Platelet *TFPI* and *ANO6* transcript levels are expressed as a ratio relative to transcripts for the housekeeping gene *PPIA*. Platelet FV levels are expressed in amounts per mg of total platelet protein. The median [range] of values, with numbers tested and p values, compare the findings for platelet function disorder and control participants.

Abbreviations: *ANO6*, Anoctamin 6; PFD, platelet function disorder; *PPIA*, peptidylprolyl isomerase A*TFPI*, tissue factor pathway inhibitor gene.

### 3.3 Associations with bleeding

**FIGURE 4:**
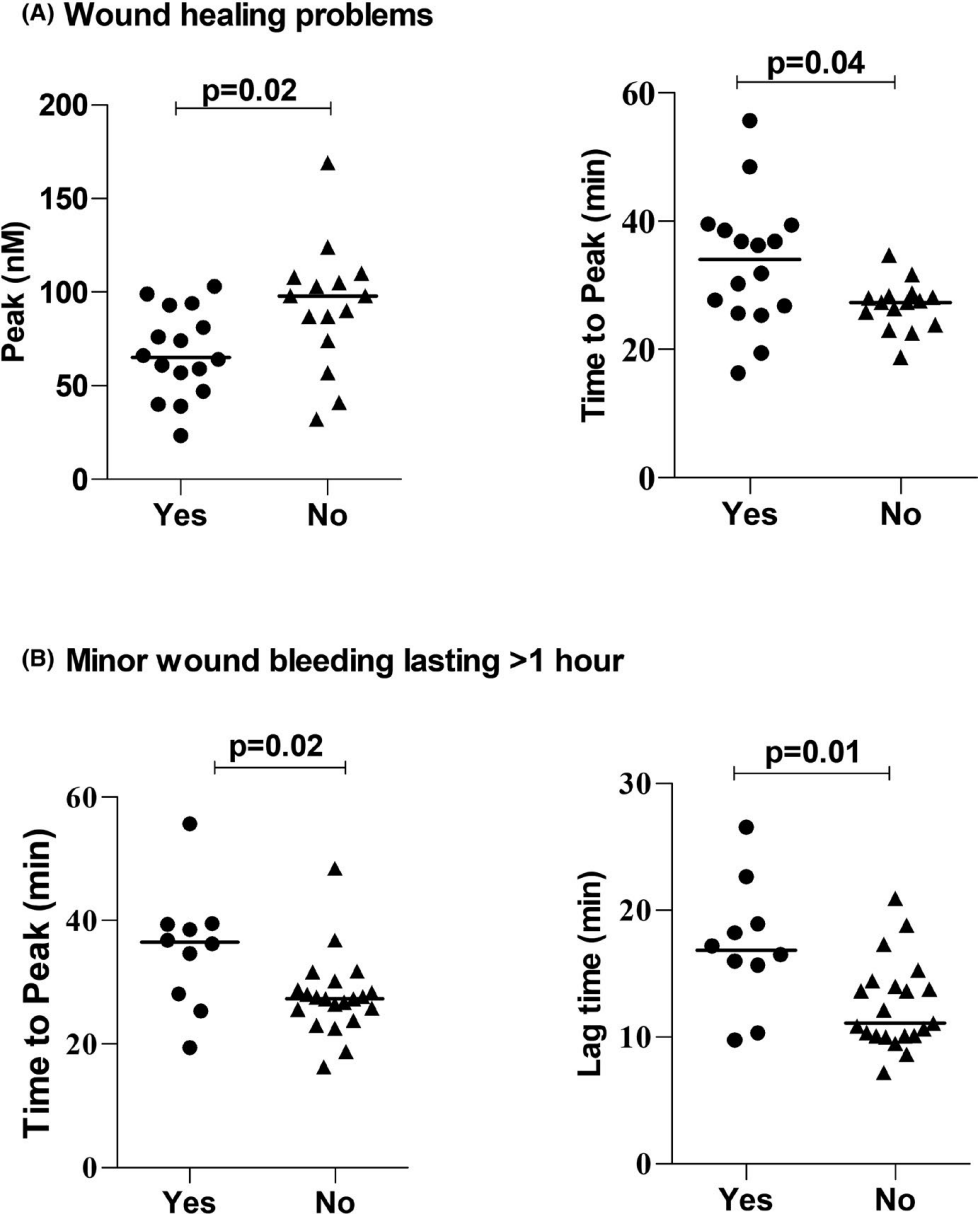
Comparisons of platelet‐rich plasma thrombin generation endpoints that were significantly different for platelet function disorder participants with wound healing and bleeding problems. Panels compare platelet‐rich plasma thrombin generation endpoints (abbreviations as in Figure 1) that showed significant differences for platelet function disorder participants with (Yes) or without (No) symptoms of wound healing problems A, and prolonged bleeding from minor wounds, lasting more than an hour B

Several PRP TG endpoints showed significant association to bleeding symptoms of PFD participants (Figure 4). First, the PRP from PFD participants that had wound healing problems had a significantly lower peak thrombin concentration, and a significantly longer time to peak TG, than PRP from PFD participants without wound healing problems (*P* ≤ .04; Figure 4A). Second, the PRP from PFD participants that had prolonged bleeding from minor wounds had a significantly longer time to peak TG and a longer lag time than PRP from PFD participants without this symptom (*P* ≤ .02; Figure 4B). None of the other TG endpoints, or other results, showed significant associations to bleeding symptoms and none showed significant association to bleeding scores.

## 4 DISCUSSION

Abnormal bleeding is a feature of PFD, and this is reflected by increased risks for bleeding, and increased bleeding scores that are higher than the bleeding scores for inherited thrombocytopenias.^3^, ^28^, ^29^ As TG studies have provided insights on other bleeding disorders, the major goal of our study was to determine if TG is impaired in a cohort with commonly encountered PFD, that included cases with nonsyndromic DGD and PFD with impaired aggregation responses to multiple agonists due to *RUNX1* mutations or other uncharacterized molecular defects. Our second goal was to determine if TG findings for PFD show significant relationships to bleeding. Importantly, we evaluated a well‐characterized PFD cohort (that included index and nonindex cases to limit bias), with increased risks for bleeding, and elevated ISTH‐BAT scores.^3^ We found significant impairments in TG in this PFD cohort, and unexpectedly, that both PRP and PPP TG were impaired. In exploring these abnormalities, we identified significant associations between PRP and PPP TG endpoints among both PFD and control participants. Although PFD‐DGD PRP TG endpoints did not show association to DG counts, the addition of polyphosphate and platelet agonists improved their PRP TG. However, even with additives, the time to peak TG for PFD‐DGD PRP remained prolonged relative to controls. We observed that our PFD cohort had increased plasma TFPI levels, and reduced platelet TFPI levels, and that their plasma TFPI levels showed significant associations to their PPP time to peak TG and lag time. Importantly, we found that several PRP TG endpoints showed relationships to PFD symptoms: PRP peak thrombin concentrations were lower, and the time to peak TG were longer, for PFD participants that had wound healing problems; and PRP TG showed an increased time to peak TG and lag time for those with prolonged bleeding from minor wounds. These interesting observations suggest that differences in TG affect hemostasis in PFD, and contribute to prolonged bleeding from minor wounds, and wound healing problems.

The major findings of our study raise interesting questions about whether studying TG would be useful for the assessment of commonly encountered PFD. The significant overlap in the TG findings for PFD and control participants suggests that TG assays would be less useful as a diagnostic tool than aggregation tests or tests for DGD. Nonetheless, it might be interesting to explore the “added value” of including TG assays in the phenotyping of PFD in future studies, including studies of larger cohorts, with diverse types of PFD. While we did not find significant associations between PPP TG endpoints and bleeding, it is possible that having defective PPP or PRP TG increases the likelihood that persons with PFD will present with bleeding problems that trigger a referral for diagnostic investigations.

Presently, few laboratories assess TG with both PRP and PPP,^30^ and we found merits to testing both sample types when assessing TG in PFD. Proteins in plasma (including TFPI, antithrombin, and fibrinogen) have important influences on TG, tested by protocols recommended for bleeding disorder assessment,^11^ without thrombomodulin and activated protein C.^18^ The important effects that plasma proteins have on TG likely underlie the strong associations that we found between PRP and PPP TG findings, for both PFD and control subjects. Our study was limited by the number of PFD cases that could be recruited and tested at our center. None of our cases had platelet FV deficiency. We did not have sufficient cases to explore for potential differences in TG findings between PFD‐DGD and other subgroups, whose findings overlapped. A larger, multicenter study would be challenging, given that few laboratories currently perform TG assays and the methods used often differ.^30^ As our PFD cohort had normal *ANO6* transcript levels, anoctamin 6 abnormalities could be highly specific for Scott's syndrome.^22^, ^23^

Previous studies have suggested that the deficiency of DG ADP or polyphosphate underlies the defective TG in syndromic or nonsyndromic forms of DGD,^1^, ^8^ however, prior studies did not evaluate for PPP TG abnormalities. While our findings for tests with additives suggest that DG ADP and polyphosphate deficiencies influence TG, adding polyphosphate and multiple agonists failed to correct all PFD‐DGD PRP TG endpoints. This could be because the ability of PFD‐DGD PRP to support TG was also influenced by the TG potential of their plasma. We postulate that the similar severity of DGD, among our PFD‐DGD participants, may be the reason why their DG counts were not a significant predictor of their PRP TG. Taken together, our new observations suggest that the TG abnormalities in PFD‐DGD are complex, with contributions from the loss of DG contents and from plasma. The underlying molecular defect for our cases with PFD‐DGD was not revealed by exome sequencing^3^ and none appeared to have αδ‐storage pool deficiency as their platelet FV was normal. An older study, that assessed TG with purified proteins and not plasma, and included cases with Hermansky Pudlak syndrome (HPS), noted that cases with HPS had more severe DGD and more severe impairments in TG than other DGD cases.^8^ As HPS is quite uncommon in our region, we were unable to include HPS cases in our study.

Normally, plasma and platelets respectively contain 90% and 10% of the TFPI in blood.^31^, ^32^ We noted that relative to controls, our PFD participants had increased plasma TFPI (which might increase bleeding) yet lower platelet TFPI levels (which might reduce bleeding), and normal platelet *TFPI* transcript levels. Nonetheless, their TFPI levels did not show associations with bleeding. While 90% of plasma TFPI is a truncated form, with reduced anticoagulant activity, platelet TFPI (which is synthesized by megakaryocytes^33^ ) is full‐length TFPIα, with equivalent anticoagulant function to recombinant full‐length TFPIα.^34^ Platelet TFPI is evolutionarily conserved, and it is thought to limit intravascular thrombus growth and modulate bleeding.^35^, ^36^ Within resting platelets, TFPI shows a diffuse distribution, unlike granule proteins.^31^ High plasma TFPI levels, like we observed in our PFD participants, have also been reported in other bleeding disorders.^20^ The positive associations that we found between plasma TFPI levels, and the TG endpoints of time to peak and lag time for PFD participants, suggest that the increased plasma TFPI levels in our PFD participants contribute to their impaired PPP TG. The associations between PFD plasma TFPI and PPP TG endpoints resemble those reported for hemophilia, factor V deficiency, QPD, and bleeding problems of unknown cause.^17^, ^20^, ^37^, ^38^

There has been growing interest in evaluating TG in bleeding disorders for novel insights on bleeding severity (reviewed in^10^). Our study is the first to report associations between TG findings and bleeding for PFD, which were significant for PRP but not PPP samples. While we did not find significant associations between TG endpoints and challenge‐related bleeding for our PFD cohort, among patients undergoing cardiac surgery, reduced preoperative PRP and PPP ETP and peak thrombin concentrations, and lower platelet counts, are associated with greater operative blood losses.^39^ The observation that desmopressin therapy for PFD improves platelet‐dependent TG,^9^ is intriguing as the challenge‐related bleeding reported by our PFD participants mainly occurred before they were diagnosed and not when they received desmopressin to reduce challenge‐related bleeding.^3^

We conclude that commonly encountered PFD are associated with defects in both PRP and PPP TG, with delayed and reduced PRP TG showing interesting associations with wound healing and bleeding problems.

## CONFLICTS OF INTEREST

SA Smith and JH Morrissey are inventors on patents and patent applications covering medical uses of polyphosphate and polyphosphate inhibitors. The rest of the authors declare that they have no real or perceived conflicts of interest to disclose.

## AUTHOR CONTRIBUTIONS

T. Sharma, J. Brunet and S. Tasneem designed and conducted experiments, analyzed and interpreted the findings, prepared figures and led the manuscript writing with CPM Hayward, who supervised the study. SA Smith and JH Morrissey provided polyphosphate, designed experiments with polyphosphate and edited the manuscript.

## Supporting information

Supplementary Material

## Data Availability

The data that support the findings of this study are available on request from the corresponding author. The data are not publicly available due to privacy or ethical restrictions.
