# Supplementary material for: Thrombin generation abnormalities in commonly encountered platelet function disorders
Source: Int J Lab Hematol. 2021 Jun 29;43(6):1557–65. doi: 10.1111/ijlh.13638 (PMC8599625; doi:10.1111/ijlh.13638)
Supplement: Supplementary file 1 — Supplementary Material [file IJLH-43-1557-s001.docx]

**Table S1. Summary of details for platelet function disorder participants, organized into subgroups.** Participants are shown by subgroups, that included platelet function disorders (PFD) due to *RUNX1* haploinsufficiency (PFD-RUNX1), PFD due to non-syndromic dense granule deficiency (PFD-DGD) and PFD with impaired aggregation responses to multiple agonists due to uncharacterized molecular causes (PFD-OTH). FV indicates factor V, TFPI indicates tissue factor pathway inhibitor, *ANO6* indicates *Anoctamin 6*, M indicates male, F indicates female.

|  | | | | | | Samples tested by ELISA | | | Samples tested for transcript levels analysis | | Samples tested for Thrombin Generation Assay experiments | | |
| --- | --- | --- | --- | --- | --- | --- | --- | --- | --- | --- | --- | --- | --- |
| Anonymized Code (family-generation-number) | Age  (years) | Sex | ISTH-BAT scores | Index (I) or non-index (N) case | Dense granule count | Platelet FV | Plasma TFPI | Platelet TFPI | *TFPI* transcript level analysis | *ANO6* transcript levels analysis | Platelet Rich Plasma  (PRP) | Platelet Poor Plasma  (PPP) | PRP with added agonists and/or polyphosphate |
| PFD-RUNX1 | |  |  |  |  |  |  |  |  |  |  |  |  |
| 1-2-2 | 49 | F | 15 | I | 6.0 | X | X | X | X | X | X | X | - |
| 1-1-1 | 73 | M | 9 | N | 4.0 | X | X | X | X | X | X | X | - |
| 1-3-4 | 22 | F | 8 | N | 5.3 | X | X | X | X | X | X | X | - |
| 1-2-1 | 46 | M | 8 | N | 4.3 | X | X | X | X | X | X | X | - |
| 1-2-3 | 41 | M | 9 | N | 4.3 | X | X | X | X | X | X | X | - |
| 8-1-1 | 64 | F | 17 | I | 4.5 | X | X | X | X | X | X | X | - |
| PFD-DGD |  |  |  |  |  |  |  |  |  |  |  |  |  |
| 5-2-2 | 34 | F | 10 | I | 1.8 | X | X | X | X | X | X | X | X |
| 6-1-2 | 81 | F | 16 | I | 1.2 | X | X | X | X | X | X | X | X |
| 6-3-2 | 31 | M | 4 | N | 0.9 | X | X | X | - | - | X | X | - |
| 7-1-1 | 68 | F | 13 | I | 2.0 | X | X | X | X | X | X | X | X |
| 7-1-2 | 64 | F | 9 | N | 1.7 | X | X | X | X | X | X | X | X |
| 11-1-1 | 49 | F | 8 | I | 1.2 | X | X | X | X | X | X | X | X |
| 12-1-1 | 76 | F | 10 | I | 2.3 | X | X | X | X | X | X | X | X |
| 17-1-1 | 42 | F | 13 | I | 2.0 | X | X | X | X | X | X | X | - |
| 23-1-1 | 63 | F | 15 | I | 1.2 | X | X | X | X | X | X | X | X |

|  | | | | | | Samples tested by ELISA | | | Samples tested for transcript levels analysis | | Samples tested for Thrombin Generation Assay experiments | |
| --- | --- | --- | --- | --- | --- | --- | --- | --- | --- | --- | --- | --- |
| Anonymized Code (family-generation-number) | Age  (years) | Sex | ISTH-BAT scores | Index (I) or non-index (N) case | Dense granule count | Platelet FV | Plasma TFPI | Platelet TFPI | *TFPI*  transcript levels | *ANO6* transcript levels, | Platelet Rich Plasma (PRP) | Platelet Poor Plasma (PPP) |
| PFD-OTH |  |  |  |  |  |  |  |  |  |  |  |  |
| 2-1-3 | 67 | F | 15 | I | 5.0 | X | X | X | X | X | X | X |
| 2-2-1 | 44 | F | 6 | N | 6.0 | X | X | X | X | X | X | X |
| 2-3-1 | 23 | F | 11 | N | 6.8 | X | X | X | X | X | X | X |
| 2-3-2 | 20 | M | 4 | N | 5.0 | X | X | X | X | X | X | X |
| 3-1-1 | 58 | F | 18 | I | 7.0 | X | X | X | X | X | X | X |
| 3-2-1 | 28 | M | 6 | N | 5.7 | X | X | X | X | X | X | X |
| 4-2-1 | 39 | F | 10 | I | 7.0 | X | X | X | X | X | X | X |
| 9-2-1 | 33 | F | 6 | I | - | X | X | X | X | X | X | X |
| 10-1-1 | 23 | M | 4 | I | 7.6 | X | X | X | X | X | X | X |
| 13-1-1 | 73 | M | 3 | I | 6.0 | X | X | X | X | X | X | X |
| 14-1-1 | 64 | F | 12 | I | 5.3 | X | X | X | X | X | X | X |
| 16-1-1 | 68 | F | 17 | I | 7.0 | X | X | X | X | X | X | X |
| 19-1-1 | 65 | F | 9 | I | 6.5 | X | X | X | X | X | X | X |
| 20-1-1 | 31 | M | 3 | I | 7.0 | X | X | X | X | X | X | X |
| 21-1-1 | 80 | F | 20 | I | 4.9 | X | X | X | X | X | X | X |
| 22-1-1 | 60 | F | 9 | I | 8.1 | X | X | X | X | X | X | X |

**Figure S1. Associations between the TG endpoints of PFD and control samples.** Associations between subjects’ platelet rich plasma and platelet poor plasma thrombin generation endpoints are shown for the 31 PFD and 40 control participants for the TG endpoints of: endogenous thrombin potential (ETP, nM*min), peak thrombin concentration (Peak, nM), time to peak thrombin generation (Time to Peak, min), and lag time (min). R^2^ and p values are shown.

**Figure S2. Associations between the TG endpoints of PFD-DGD samples.** Associations between subjects’ platelet rich plasma and platelet poor plasma thrombin generation endpoints are shown for the 9 PFD-DGD participants for the TG endpoints of: endogenous thrombin potential (ETP, nM*min), peak thrombin concentration (Peak, nM), time to peak thrombin generation (Time to Peak, min), and lag time (min). Numerical values shown beside each subject’s colored symbol indicates their platelet dense granule count, which did not show significant relationships to their PRP TG endpoints (R^2^ 0.001-0.20, p values ≥0.23).

**Table S2. Associations between the thrombin generation endpoints for platelet rich plasma and protein and transcript levels for platelet function disorder participants.** Thrombin generation endpoints, including ETP (endogenous thrombin potential) were tested for associations with: platelet and plasma TFPI levels, platelet FV levels, *TFPI/PPIA* transcript ratios, and *ANO6/PPIA* transcript ratios, using data for all PFD participants. For each analysis, the R^2^ value, p-value, and information on whether the association was positive or negative, is shown.

|  |  | | | |
| --- | --- | --- | --- | --- |
|  | **ETP**  **(nM*min)** | **Peak thrombin concentration (nM)** | **Time to peak**  **(min)** | **Lag time**  **(min)** |
| Platelet TFPI levels (ng/mg platelet protein) | R^2^=0.05  p=0.22  negative | R^2^=0.03  p=0.25  negative | R^2^=0.002  p=0.81  positive | R^2^=0.10  p=0.08  positive |
| Plasma TFPI levels (pg/ml) | R^2^=0.03  p=0.38  negative | R^2^=0.05  p=0.21  negative | R^2^=0.02  p=0.43  positive | R^2^=0.05  p=0.20  positive |
| Platelet FV levels (μg/ml) | R^2^=0.13  p=0.06  positive | R^2^=0.08  p=0.14  positive | R^2^=0.09  p=0.12  negative | R^2^=0.10  p=0.10  negative |
| *TFPI/PPIA* transcript ratios | R^2^=0.04  p=0.29  positive | R^2^=0.03  p=0.39  positive | R^2^=0.10  p=0.09  negative | R^2^=0.13  p=0.05  negative |
| *ANO6/PPIA* transcript ratios | R^2^=0.05  p=0.51  negative | R^2^=0.02  p=0.40  negative | R^2^=0.07  p=0.15  negative | R^2^=0.07  p=0.15  negative |
